# Aphasia-Diagnostic Challenges and Trends: Speech-Language Pathologist’s Perspective

**DOI:** 10.12669/pjms.37.5.2314

**Published:** 2021

**Authors:** Faiza Badar, Sajida Naz, Nazia Mumtaz, Muhammad Naveed Babur, Ghulam Saqulain

**Affiliations:** 1 Ms. Faiza Badar, MS (SLP) Manager, Rehabilitation Department, Shifa International Hospital, Islamabad, Pakistan; 2 Dr. Sajida Naz, PhD (Trauma Psychology) Assistant Professor, Department of Behavioral Sciences, Fatima Jinnah Women University, Rawalpindi, Pakistan Adjunct Assistant Professor IIRS, ISRA University Islamabad, Pakistan; 3 Dr. Nazia Mumtaz, PhD (Rehabilitation Sciences), Head of Department of Speech Language Pathology, Faculty of Rehab & Allied Health Sciences, Riphah International University, Islamabad, Pakistan; 4 Dr. Muhammad Naveed Babur, PhD (Rehabilitation Sciences) Dean & Professor, Faculty of Allied Medical Sciences, ISRA University, Islamabad, Pakistan; 5 Dr. Ghulam Saqulain, F.C.P.S (Otorhinolaryngology), Head of Department & Associate Professor, Department of Otorhinolaryngology. Capital Hospital PGMI, Islamabad, Pakistan

**Keywords:** Aphasia, Assessment, Speech language pathologists, tools

## Abstract

**Objectives:**

To explore current aphasia assessment practices and barriers among Pakistani speech language pathologists.

**Methods:**

Descriptive study design with qualitative parameters was used. Participants were identified using purposive sampling over a period of eight months from 1st December 2018 to 31st July 2019. Sample comprised of ten speech-language pathologists with least five years’ experience of working with aphasic clients from four major cities of Pakistan including Islamabad, Karachi, Lahore and Peshawar. Study included in depth interviews using a self-structured interview guide with probe questions. Data recorded was transcribed and thematic analyses were drawn manually.

**Results:**

Thematic analysis revealed that most Speech language pathologists rely heavily on informal assessment techniques. With no aphasia assessment tool available in Urdu language, no consensus as to the optimal evaluation strategy or tool for aphasia assessment was noted. However, need for such tool was highlighted by all participants. Hence, non-availability of standardized and culturally appropriate assessment tool in “Urdu” language turned out to be the major barrier in adopting formal assessment for aphasic clients, while time consumed in formal testing remained second most reported issue.

**Conclusion:**

There is a dire need of quick aphasia assessment tool in Urdu language with established psychometric properties and culturally appropriate norms.

## INTRODUCTION

South Asia harbors thickly populated countries with Pakistan being fifth most populous country around the globe.^1^ Also, a trend toward urbanization has resulted in socioeconomic changes with better control of infectious diseases leading to decreased mortality and increased life span. With this change, probability of risk towards illnesses of old age including cardiac disease and episodes of stroke have multiplied. Aggravated by higher rates of diabetes and blood pressure and that too at younger age, the incidence of small-artery occlusion ischemic stroke has increased among South Asians^2^, with trends on stroke incidence globally indicating a 42% reduction in high income countries and an alarmingly 100% increase in low to middle income countries.^3^

There is limited population-based data available on epidemiology of stroke from developing countries of South Asia including Pakistan. Hence awareness regarding risk factors and management of stroke among general public and caregivers is very limited and unreliable.^4^

Aphasia is one of common post stroke complications and has a noticeable effect on quality of life of the patients.^5^ It can result in inability to properly comprehend or formulate language depending on the area effected by neurological trauma.^6^ Clinical guide lines for stroke management published by National stroke foundation in 2010 recommended that patients with suspected aphasia should be formally assessed by a speech language pathologist to rule out presence of aphasia.^7^ Timely detection of aphasia in stroke patients is crucial for timely treatment planning and establishing a prognosis. A number of screening tools are used for detection of aphasia in post stroke patients, with many lacking required psychometric properties.^5^

Pakistan is a developing country with large population living below poverty line with a study from Lahore revealing the fact that 54.3% of stroke survivors developed aphasia.^8^ Difficulty and deficit in communication is common occurrence as a result of neurological insult with primary physicians attending such patients are first to note such deficit. The area of infarct or damage can be seen in neuroimaging reports and only in case of major involvement of language dominant area, aphasia is suspected. Only on finding communicational difficulty a speech language pathologist is consulted. Early detection, diagnosis and intervention of language deficits are vital in maximizing rehabilitation gains. Hence, a screening test can always be a helpful tool in the early identification and appropriate referral of individuals with possible underlying language problems. Formal and detailed language evaluation by speech/language pathologist is crucial to find out type of aphasia and levels of impairment. Standardized evaluation also helps in planning therapy, and assess patient’s prognosis. Ideally this assessment must aim to evaluate all components of language individually and in depth. Components of aphasia tests include assessments of auditory comprehension, spontaneous speech, ability to repeat, naming skills, reading, and writing skills. A number of assessment tests are used worldwide to evaluate patients suspected with aphasia to determine presence or absence, type and degree of aphasia. Research and systematic reviews conducted on existing aphasia screens aimed to find out accuracy of evaluation, reliability and feasibility of using these tests.^5^

In acute phase a patient with severe language impairment finds it difficult to cooperate and participate in lengthy language assessments. Test tools such as WAB (Western aphasia battery) and BDAE (Boston diagnostic aphasia examination) are time consuming.^9^ Therefore, this study was designed to explore current aphasia assessment practices and barriers among Pakistani speech language pathologists. This study may help access the possibilities to develop indigenous measures and fill the gap in clinical and research practices.

## METHODS

The current research was an exploratory descriptive study, employing purposive sampling technique with qualitative parameters, conducted following approval of Institutional Review Board/ Ethical Committee of Shifa International Hospital vide Ref. No. 1199-475-2018 dated 17^th^ January, 2019. Study was conducted in four cities of Pakistan including Islamabad, Karachi, Lahore and Peshawar over a period of eight months from 1st December 2018 to 31st July 2019. Sample included ten speech language pathologists having at least five years of working experience with aphasic clients.

A structured interview guide was developed to explore the perspective of speech language pathologists on diagnostic and therapeutic challenges they faced managing aphasic clients. The guide contained easy to comprehend questions based on themes including: current assessment tools in use, barriers in use of formal testing and need for standardized assessment tools for aphasic clients. Furthermore, the probes of each domain were built in to the relevant sections of this interview guide, which served to steer the discussion into the domain areas without leading the respondents. The researcher provided the least necessary direction for the respondents to focus on the domains for exploration of multi-level barriers with reference to formal assessment, and allowed the respondents to express their views at length and in an uninhibited manner.

**Fig.1:**
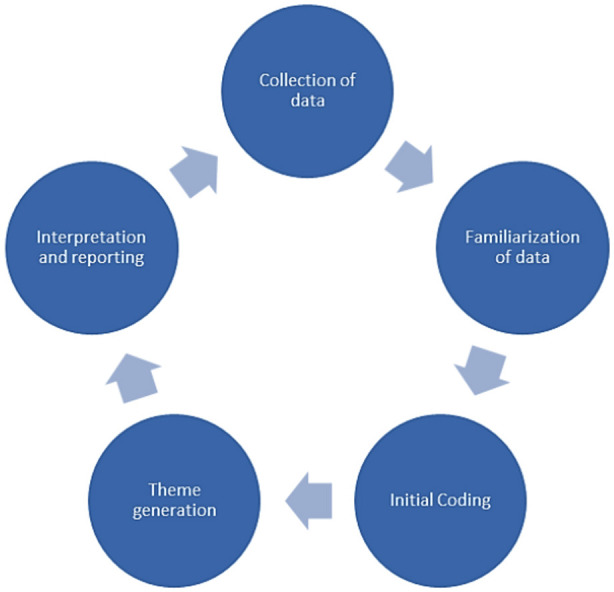
Steps of Thematic Study.

Study was conducted after obtaining consent from speech therapists selected as study sample following steps as depicted in Fig.1. Questions about characteristics of an ideal assessment tools were put forward to extract extensive exhaustive answers and probes were resorted to when extensive information was sought and voice record obtained. Data was subjected to content analysis and thematic analyses were drawn manually using coding.

## RESULTS

Majority of the study population was female 8(90%), working in public setups 5(50%) and having experience of 5 to 10 years 6(60%) (Table-I).

**Table-I:** Demographics of Study Population (n=10).

Variable	Category	n(%)
Gender	Male	2(20)
	Female	8(80)
Setup	Public	5(50)
	Private	4(40)
	Semiprivate	1(10)
Experience	5	3(30)
	5 to 10	6(60)
	>10	1(10)
Location	Islamabad	4(40)
	Lahore	2(20)
	Peshawar	2(20)
	Karachi	2(20)

Outcomes drawn from thematic analysis (reference) were categorized in four principles themes extracted from the interviews (Table-II). Re-analysis of themes showed considerable consistency in terms of measures and assessment methods used by the respondents. Results revealed that there was no Standardized Aphasia Assessment Tool available in Urdu language. Time consumed in translating available test in native language was also a problem faced and reported by speech language pathologists. The results can draw the attention of researchers towards this domain and highlight need of aphasia assessment tool in the native language.

**Table-II:** Results of Thematic Analysis: Themes & Characteristics.

S. No.	Themes	Characteristics
1	Current assessment practices	Informal, translated tools, components from language tools, self- developed checklists
2	Barriers in formal assessment	Non-availability of culturally appropriate tools, Non-availability of assessment tools in native language, Time constraints, Lack of cooperation from patients and attendants, financial constraints
3	Justification of tool in native language	For accurate assessment, for re-evaluation to measure results of therapy, for conducting researches in aphasiology
4	Requirements of aphasia assessment tool according to local needs	Tool in native language, tool with scoring manual, tool that is easy to administer, tool that is less time consuming, tool that is not expensive and tool that is culturally appropriate.

**Fig.2:**
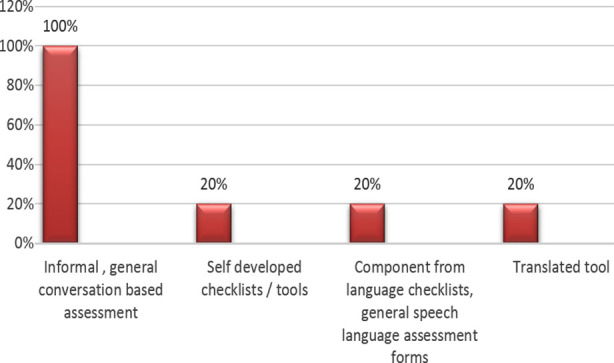
Aphasia assessment techniques being used by sample population (n=10).

Study revealed that different assessment techniques were adopted by the SLP’s for aphasia patients, however all (100%) participants used general conversation to assess aphasia. Additionally, 2(20%) each also used general speech and language form, non-standardized self-developed tools and translated versions of tools available in English (Fig.2)

## DISCUSSION

The current research probed into existing practices of aphasia assessment and barriers among Pakistani speech language pathologists related to the aphasia. Findings of this study are consistent with studies carried out in countries with similar geographic attributes. A systematic review of language tests used for diagnosing aphasia by Rohde A et al. concluding that accurate diagnosis of post-stroke aphasia was essential for management. With a number of assessment tools noted to be in use for evaluating aphasia, however many of in use tools lacked validation, or the procedure for standardization was found to be inadequate, highlighting need for a robust test for diagnosis of aphasia in stroke population.^10^

In the current study, respondents highlighted a number of issues related to aphasia diagnosis and proposed that although a standardized aphasia test is useful in providing with structured guidelines, there is still need for an indigenously informed tool that addresses local needs and clinical experiences according to the Pakistani culture. Respondents identified range of difficulties associated with aphasia diagnosis such as: absence of a standardized population-specific aphasia tool that meets local language and cultural needs, aphasia assessment should be dynamic including observation and informal conversation data collected online of components mentioned in formal test batteries. Not having a culturally valid assessment tool for aphasic population restricts speech language pathologists to carry out researches in aphasiology as evident by information collected from participants of current study.

Participants of study highlighted that informal procedures when used for assessment are subject to bias and lag behind in terms of accuracy and therefore there should be a standardized aphasia assessment tool in our national language “Urdu”. Speech language pathologists do not feel adequately trained in assessment of aphasia, they reported that assessment tools and administration of these tools was not part of their curriculum, they are only accustomed with names of standardized tools but they have never seen or used them. “It is very expensive to import an aphasia assessment battery and then it will be in English language so I don’t see any logic in having it especially when we are making good assessments using informal assessments.” a respondent affirmed.

These findings are largely consistent with those of Vogel AP et al. in the study conducted in Australia and New Zealand on a sample of 174 speech pathologists practicing in acute care set ups, regarding most frequently used aphasia assessment tool showed that majority (70%) preferred to go for informal assessments as compared to the formal evaluations most of the time utilizing their self-developed screening tools, sub tests or non-standardized assessments.^11^ Similarly in a local study by Azhar A et al. 50% of the sample used self-developed tests which they were using in their setups.^12^ Also due to a number of pitfalls in translating a tool into local language, development of a new test in a local language is preferable^13^, with most translated tools not suitable for assessment of aphasia in local context.^14^

Worrall LS et al. studied frequency of aphasia tests usage on a sample of 14 speech therapists in their Australian study and concluded that use of informal assessments is third commonly used method for aphasia assessment. Therapists who participated in this particular study suggested that test developers must focus on ensuring utility of assessment tools. Clarity of instructions in manual and pictures used in assessment tests must be qualitative.^15^ Authors of study which was conducted on a variety of rehabilitation professionals including physical, occupational and speech pathologists regarding use of standardized assessment tests for assessment of post stroke patients concluded that speech professionals showed the lowest adherence levels among all disciplines when it came to use of standardized assessment tools.^16^

It is expected that speech language pathologists translate available tests in Urdu language when they are evaluating patients with suspected aphasia but research indicate that word to word translation of an available assessment tool in another language is not suitable reason being that there is no similarity between words and syntax structure of any two languages.^17^ Aphasia is a language disorder in which receptive language and expressive language components are assessed in detail, language barriers can result in false positive or false negative thus effecting treatment plan.^18^ An Australian study by Roger & Code to measure validity of assessment when a test is translated by an interpreter to evaluate patient with aphasia revealed that content validity was often decreased during test administration or during the process of communicating responses to speech pathologist by interpreter.^19^

Checklists for screening purpose are developed and in use at departmental levels but their reliability and validity are questionable. Hence, speech and language pathology professionals are in need of an assessment tool which is not lengthy and had culturally appropriate content. Interviews highlighted concern of speech language pathologists, regarding affordability issues faced by their patients and families, it appears to be major reason for loss of follow up, so any assessment tool requiring more than one sitting can raise financial concerns for clients and their families.

A web-based survey on current aphasia rehabilitation practices of speech-language pathologists revealed somewhat similar challenges some of which included inflexible funding models for aphasia therapy resulting in limited follow ups. Study also concluded that practitioner feel they are not well equipped with counseling skills which is frequently required during rehabilitation of patients with aphasia. Respondents of under discussion study when reporting challenges faced during practice came up with lack of resources (time, space, materials) as a major challenge to effective service provision.^20^

Development of reliable and valid assessment test in native language will result in better assessment and reassessment for measuring improvement in aphasic clients over a period of time. Availability of assessment tool will also pave the way for professionals in related field to conduct research in this domain in future.

Speech pathologist frequently deals with cultural and linguistic diversity resulting in considerable challenges when it comes to providing equal services to all clients. When facing language barriers speech practitioners are often forced to call on primitive personnel to act as interpreters or to translate assessment test for patients. A study conducted in south Africa explored whether linguistic complexity after translation of the Western Aphasia Battery (WAB) test by five isiZulu language speakers from English to isiZulu was affected or not. Results were indicative of noticeable differences in the translations, with most differences relating to vocabulary and semantics. Hence, we need to understand clinical implications of using translated material for assessment of speech and language in multilingual contexts.^21^

This thematic analysis using descriptive study design has contributed to local literature by added important barriers to current aphasia practices and highlighted the need of an Urdu language aphasia assessment tool for further research.

### Limitations of the study

Approaching and getting appointments from the participants and recording of the interviews were the main hurdles.

## CONCLUSIONS

There is a dire need of quick aphasia assessment tool in Urdu language with established psychometric properties and culturally appropriate norms. Accurate diagnosis is crucial because treatment strategy is based on results of assessment. Currently there is no aphasia assessment test with proven validity and reliability in Urdu language.

### Authors Contribution

**FB** did the data collection, analysis and manuscript writing and is responsible for the integrity of the work.

**SN** was responsible for conception & designing of research.

**NM** did the methodology and literature review.

**MNB** did the data interpretation.

**GS** did the critical revision of the article.
